# A Comprehensive Study of the Use of Cu(I)/4,4’-Dicarboxy-2,2’-biquinoline Complexes to Measure the Total Reducing Capacity: Application in Herbal Extracts

**DOI:** 10.3390/molecules201219855

**Published:** 2015-12-14

**Authors:** Hariane R. Manoel, Horacio D. Moya

**Affiliations:** CEPES (Centro de Estudos, Pesquisa, Prevenção e Tratamento em Saúde), Faculdade de Medicina da Fundação do ABC, Av. Príncipe de Gales 821, Santo André 09060-650, Brazil; harirezende@hotmail.com

**Keywords:** 4,4′-dicarboxy-2,2′-biquinoline acid, Cu(II), total reducing capacity, Brazilian medicinal plants

## Abstract

A method for the determination of total reducing capacity (TRC) based on the reduction of Cu(II) to Cu(I) by antioxidants in a buffered solution (pH 7.0) containing 4,4′-dicarboxy-2,2′-biquinoline acid (BCA) was developed. Absorbance values at 558 nm characteristic of the Cu(I)/BCA complexes formed were used to determine the TRC of aqueous extracts of twelve Brazilian plants. The TRC values obtained with the suggested method correlated well with values obtained using the 2,2-diphenyl-1-picrylhydrazyl (DPPH) method (r^2^ = 0.959). They were also compared with the total polyphenol content (using the Folin-Ciocalteu reagent) and the good agreement (r^2^ = 0.919) indicates that the polyphenols should be responsible for this reducing capacity. The method proposed here (and successfully applied in plant extracts) can be used to measure the TRC of aqueous samples derived from other plants (e.g., teas, juices, beers and wines) and even in biological samples (e.g., serum, urine and follicular fluid). To achieve a structure-activity relationship of the proposed reaction, the reduction capability of 25 standard antioxidants (phenolic derivatives, flavonoids, stilbenoids, vitamins, *etc.*) was individually evaluated and the apparent molar absorptivity values (at 558 nm) obtained were compared and discussed.

## 1. Introduction

There is a growing interest in increasing the reducing capacity of diet components, leading to the development of different methods for quantifying this activity in fruits, fruit juices, wine, beer, tea and herbal extracts [1]. Other methods have been proposed and used in serum and plasma samples after ingestion of foods with potential antioxidant activity [2].

However, the chemical complexity of antioxidants in these samples makes their separation and identification by routine methods difficult. Therefore, it is always advisable to use more economical methods which allow the quantification of the total reducing capacity in a quick but reliable approach.

The methods for determining the reducing capacity can be mechanistically divided into hydrogen atom transfer (HAT) and single electron transfer (SET) ones. The first group includes Trolox equivalent antioxidant capacity (TEAC), total radical trapping antioxidant potential (TRAP) and oxygen radical absorbance capacity (ORAC). The SET type methods include ferric reducing antioxidant power (FRAP) and copper reducing antioxidant capacity (CUPRAC) as the most representative [1,2].

4,4′-Dicarboxy-2,2′-biquinoline acid (BCA) is a weak organic acid (pKa_1_ = 1.87; pKa_2_ = 2.85) derived from quinoline. It is a selective complexing agent for Cu(I) in aqueous media and has been used for the direct determination of copper in soil [3] and indirect determination of proteins [4], sugars [5], ascorbic [6] and uric [7] acids. The addition of proteins, reducing sugars, ascorbic and uric acids in aqueous BCA solution (pH ≥ 7.0) reduces Cu(II) to Cu(I), which then forms a violet complex with BCA whose absorbance values taken at 558 nm (ε_558nm_ = 8.7 × 10^3^ mol^−1^·L·cm^−1^) [8] are proportional to the concentration of these analytes.

The reduction reaction of Cu(II) in BCA medium (pH =7.0; maintained with ammonium acetate) has been utilized by our research group for the development of analytical methods for the indirect determination of drugs and polyphenols in different samples [9,10,11,12]. In the present study an alternative to SET type methodology for the quantification of the total reducing capacity based on the Cu(II)/Cu(I) reduction reaction is proposed.

First, twenty five standard antioxidants (phenolic derivatives, flavonoids, stilbenoids and vitamins) were individually evaluated by determining their apparent molar absorptivity values at 558 nm (A_558nm_). Then, the reduction capacity of each compound was calculated by comparing these values with the apparent molar absorptivity of an ascorbic acid (AA) solution (a standard antioxidant compound) also measured at 558 nm in the same experimental conditions. Finally, the reduction capacity values obtained were discussed with regards to the molecular structure of each antioxidant.

Aqueous extracts of Brazilian medicinal plants were chosen to verify the applicability of the proposed method in natural samples. Since herbal extracts contain several antioxidant compounds (especially polyphenols) the addition of aliquots of these samples to a solution containing Cu(II) also promotes its reduction to Cu(I), which in the presence of BCA (pH ≥ 7.0) forms the Cu(I)/BCA violet complex. The A_558nm_ values of five diluted solutions of each extract were compared with the A_558nm_ values of a curve obtained with an AA standard solution and used to quantify the reducing capacity of these samples and also to express these values as AA equivalents. The total reducing capacity of these same extracts were compared with the values obtained with the 2,2-diphenyl-1-picrylhydrazyl (DPPH) method [13,14] and with the total polyphenolic content quantified using the Folin-Ciocalteu (FC) reagent [15].

## 2. Results and Discussion 

### 2.1. Determination of the Reduction Capacity of Some Antioxidant Compounds with the Proposed Method

In this study Cu(I) was obtained by reduction of Cu(II) in BCA medium by several antioxidants (AOs). The reaction can be described by Equation (1). As mentioned before [9,10] the best experimental conditions (Cu(II):BCA in a 1:3 ratio) were achieved in a solution containing 0.5 × 10^−3^ M Cu(II), 1.5 × 10^−3^ M BCA and 0.8 M ammonium acetate (pH 7.0). At higher concentrations (e.g., 1.0 × 10^−3^ M Cu(II) and 3.0 × 10^−3^ M BCA) a light green Cu(II)/BCA precipitate was formed. A decrease in the absorbance values was noted at lower concentrations (e.g., 0.05 × 10^−3^ M Cu(II) and 0.15 × 10^−3^ M BCA):

Cu(BCA)_2_^2^^−^ + AOs → Cu(BCA)_2_^3^^−^ + AOs _oxidized_ (1)

**Figure 1:**
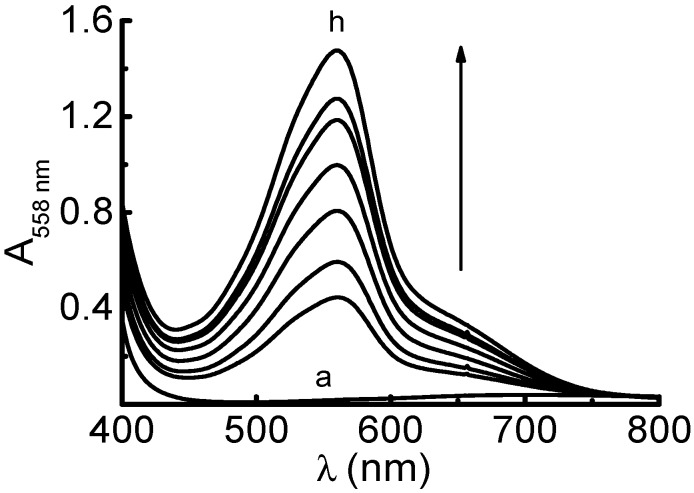
Absorption spectra of: (a) = Cu(II) 5.0 × 10^−4^ M + ammonium acetate buffer solution (pH = 7.0) 0.8 M + BCA 1.5 × 10^−3^ M; (b to h) = (a) + Ascorbic acid (0.71; 1.06; 1.41; 2.11; 2.47; 2.82 and 3.17) × 10^−2^ mg·mL^−1^, respectively. Measurements using water as reference solution.

A typical absorption spectrum of a Cu(II) solution before and after AA addition in a medium containing BCA is shown in Figure 1. AA was used as a standard antioxidant compound to compare the reduction capacity of some other antioxidant compounds (AOs) with the proposed method. This reducing ability can be expressed in AA equivalents capacity (AA_EC_) defined as the AA concentration (in 10^−3^ M), which has the same reducing capacity of a 1.0 × 10^−3^ M solution of a given AO [16]. These values can be more easily calculated by dividing the slopes (*b*) of the straight-line equation obtained from the calibration curve of any AO (A_558nm_ = *a* + *b* × C_AO_), by the “*b*” of the straight-line equation obtained from AA calibration curve (A_558nm_ = *a* + *b* × C_AA_). All values were obtained at 558 nm under the same experimental conditions [16].

**Figure 2:**
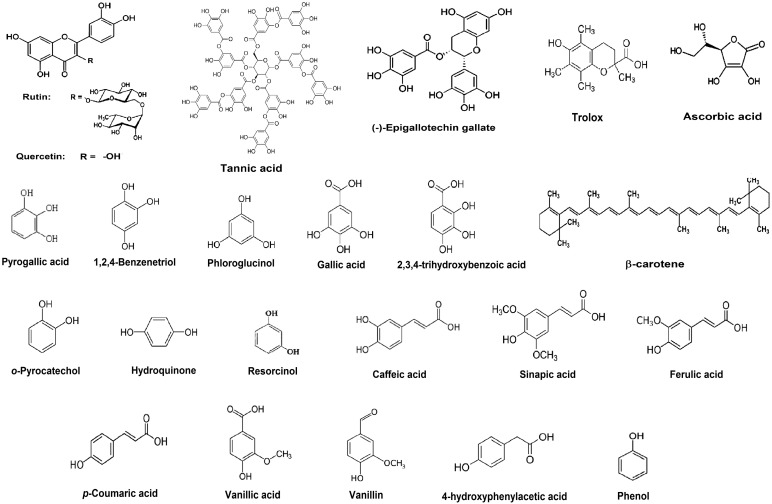
The formulas of antioxidant compounds examined with the proposed method.

Only calibration curves that showed good linearity (r^2^ ≥ 0.99) were used to calculate these “*b*” values for any AO studied. The average value of “*b*” originating from at least triplicate calibration curves (in M) of each AO was divided by the average value of “*b*” obtained from the AA calibration curves (6.73 × 10^3^) resulting in the AA_EC_ values for each AO evaluated. Table 1 shows these AA_EC_ values along with the linear range of a typical calibration curve of each AO, whose formulas are shown in Figure 2.

**Table 1:** Ascorbic acid equivalent capacity of antioxidant compounds obtained with the proposed method.

Phenolic Compound	FHG	FW (g·mol^−1^)	LR/10^−5^ (M)	b/10^3^	AA_EC_
Tannic acid	25	1701.23	0.1–0.7	259 ± 12	38.1 ± 1.7
Pyrogallic acid	3	126.11	1–4	18.7 ± 1.9	2.76 ± 0.28
1,2,4-Benzenetriol	3	126.11	0.8–2.2	24.5 ± 0.3	3.61 ± 0.05
Phloroglucinol	3	126.11	1.6–13	10.0 ± 0.4	1.47 ± 0.06
Gallic acid	3	170.12	0.1–0.7	39.4 ± 1.5	5.81 ± 0.23
2,3,4-THB	3	254.28	0.2–1.6	35.6 ± 0.2	5.24 ± 0.03
*o*-Pyrocatechol	2	100.11	0.2–1.6	36.5 ± 0.6	5.38 ± 0.09
Hydroquinone	2	110.11	1–8	14.3 ± 0.1	2.11 ± 0.01
Resorcinol	2	110.11	1.8–15	6.16 ± 0.50	0.91 ± 0.07
Caffeic acid	2	180.16	0.2–1.6	31.0 ± 0.8	4.57 ± 0.12
Sinapic acid	1	224.21	1–8	16.8 ± 0.2	2.47 ± 0.03
Ferulic acid	1	194.19	1–8	12.3 ± 0.5	1.82 ± 0.08
*p*-Coumaric acid	1	164.16	4–32	2.43 ± 0.23	0.36 ± 0.03
Vanillic acid	1	168.15	2–14	5.27 ± 0.41	0.81 ± 0.06
Vanillin	1	152.13	26–40	0.66 ± 0.06	0.10 ± 0.01
4-Hydroxyphenylacetic acid	1	152.13	12–40	0.75 ± 0.07	0.11 ± 0.01
Phenol	1	94.11	106–848	(65 ± 3) × 10^−3^	(9.6 ± 0.4) × 10^−3^
Quercetin	5	302.24	0.2–1.6	66.1± 0.7	9.74 ± 0.11
Rutin	10	610.58	0.2–1.6	41.6 ± 0.7	6.13 ± 0.11
(−)-Epigallocatechin gallate	8	458.37	0.2–1.6	65.5 ± 2.6	9.65 ± 0.38
β-Carotene	-	536.87	1–8.1	1.49 ± 0.04	0.22 ± 0.01
Trolox	1	250.29	26–40	19.2 ± 1.9	2.83 ± 0.28
Ascorbic acid	-	176.13	4–20	6.78 ± 0.46	1.00

FHG = phenolic hydroxyl group; FW = formula weight; LR and b are linear range and slopes of the calibration curves, respectively; AA_EC_ = ascorbic acid equivalent capacity; 2,3,4-THB = 2,3,4-trihydroxybenzoic acid. Data represent average and standard deviation of at least three measurements.

Tannic acid is a mixture of polygalloyl glucoses and due its higher number of free hydroxyl groups (FHGs) it has the highest AA_EC_ value, which is in agreement with theory [16].

Comparing only the trihydroxybenzene isomers it is possible to note that 1,2,4-benzenetriol (1,2,4-B) has a bigger AA_EC_ value. The presence of two FHG in the *ortho* (1,2-) arrangement in pyrogallic acid effectively facilitates the Cu(II) reduction, but the presence of another vicinal FHG (1,2,3-) decreases its reducing capacity when compared with 1,2,4-B, which is in agreement with theoretical information [17,18,19]. In phloroglucinol the symmetrical (1,3,5-) FHG distribution decreases the AA_EC_ [17,20]. In gallic acid the deprotonated carboxyl group (pKa_1_ = 4.7) [21] in the reaction medium (pH 7) increases the resonance of the benzenic ring that might be responsible for duplicating the AA_EC_ value of pyrogallic acid. The 2,3,4-trihydroxybenzoic acid (2,3,4-THB) has also a deprotonated carboxyl group (pKa_1_ = 3.0) [21], but unlike gallic acid the -COOH group is next to the three FHG. Despite this structural difference the AA_EC_ value of 2,3,4-THB (5.24 ± 0.03) is only about 10% lower than that of gallic acid (5.81 ± 0.23), as shown in Table 1. It can be inferred that for the trihydroxybenzenes the position of one -COOH group included in the benzenic ring does not profundly affect the AA_EC_ value.

Considering the dihydroxybenzenes the *ortho* position also contributes to the highest AA_EC_ value of pyrocatechol (5.38 ± 0.09; Table 1). In fact, the reducing capacity values vary with the position of the second FHG being AA_EC_ pyrocatechol > AA_EC_ hydroquinone > AA_EC_ resorcinol. The *para* position in hydroquinone appears to favour a more easy electron donation than the *meta* position in resorcinol, which increases the ability to reduce the first (2.11 ± 0.01) compared to the last (0.91 ± 0.07) [17]. Indeed, oxidation of phenols to quinones seems to be easier if two FHG are in the *para* or *ortho* positions in the aromatic rings [20].

Caffeic acid is a dihydroxylated cinnamic acid derivative and has a -CH=CH-COOH group in opposite position to the two *ortho* FHG. The -COOH group is deprotonated (pKa_1_ = 3.0) [21] under these experimental conditions. Caffeic acid has a reducing capacity 1.3 times higher than 1,2,4-B and the same considerations made above for AA_EC_ gallic acid/AA_EC_ pyrogallic acid relation could be used here. 

Three monohydroxylated cinnamic acid derivatives (HO-Ar-CH=CH-COOH) were investigated. Under the experimental conditions sinapic (pKa_1_ = 4.58), ferulic (pKa_1_ = 3.60) and *p*-coumaric acids (pKa_1_ = 4.64) [21] are all deprotonated. In sinapic acid the presence of two -OCH_3_ donating groups (Table 1) increases the AA_EC_ value 1.4 and 6.9 times when compared with ferulic and coumaric acids, respectively. In ferulic acid there is only one -OCH_3_ group. It allows the AA_EC_ value (1.82 ± 0.08) to be 5 times higher when compared with *p*-coumaric acid that has no -OCH_3_ group (0.36 ± 0.03). The *p*-coumaric acid has just one FHG and an AA_EC_ value almost 13 times smaller than caffeic acid which has two (Table 1). These values also seem to agree with theory [20,22].

Vanillic acid and vanillin have -OCH_3_ groups. Vanillic acid (pKa = 4.45) [21] is an oxidized form of the aldehyde vanillin, which has a -CHO group. When comparing these two compounds it is noted that as the acid group in vanillic acid is dissociated under the experimental conditions. The AA_EC_ increased eight-fold probably due to a resonance effect. The presence and the distance of the -COOH group attached to the benzenic ring in phenolic derivatives seems to affect the reductive capacity with the proposed method. In fact, it becomes clearer when compared with the AA_EC_ values of ferulic acid (1.82 ± 0.08), a starting compound for the production of vanillin, AA_EC_ 0.10 ± 0.01, and vanillic acid, AA_EC_ 0.81 ± 0.06 (Table 1).

Another example arises from phenol, which has just one FHG and is a very weak reducing agent (AA_E__C_ 9.6 × 10^−3^). Here again, the introduction of -CH_2_-COOH group gives the 4-hydroxyphenylacetic acid (pKa_1_ = 4.25) [21], which is 10 times (0.11 ± 0.01) more reducible than phenol.

The observations above confirm the common thought that the presence of FHGs on a benzenic ring is responsible for the reducing properties of these compounds. As expected, the number and position of FHGs, as well as the presence and distance of other radicals assume important roles in the reduction of phenolic acid derivatives. 

The same behaviour seems to occur with flavonoids, which is another class of antioxidant compounds that have great reducing activity (Table 1). Quercetin and rutin have the same aglycone. However, rutin (a quercetin disaccharide derivative) has an AA_EC_ value 1.6 times lower than the quercetin. On the other hand, (−)-epigallocatechin gallate (an ester of gallic acid with epigallocatechin) has an AA_E__C_ 9.65 ± 0.38, which is 1.7 fold than the AA_EC_ for gallic acid (5.81 ± 0.23). This increase can be attributed to the acid esterification with catechin, which itself increases FHGs from 3 to 8.

β-Carotene (provitamin A) does not contain an-OH group but showed AA_EC_ twenty two times higher than phenol. Based on the fact the stock solution of this compound was prepared in 40:10 (*v*/*v*) acetone–water mixture, that could lead to the conclusion that the proposed method could be used in less hydrophilic solutions (Table 1).

Trolox, a water soluble analogue of vitamin E, can also be used to quantify the reduction capacity with the proposed method. AA_EC_ value is almost three times higher than AA (Table 1). However, it is almost 100 times more expensive.

Finally, it can be observed from Table 1 that the higher the AA_EC_ value for a given compound the lower the working linear range, which indirectly means higher sensitivity. This indicates that small quantities of this compound with high AA_EC_ value will greatly increase the value of the total reduction capacity of the sample measured with the proposed method.

For these same antioxidant compounds it worth noting that a similar trend was observed considering the reduction reaction of Fe(III) to Fe(II) in aqueous solution containing the 3-hydroxy-4-nitroso-2,7-naphthalene disulfonic acid (pH 8.0; TRIS). This trend is demonstrated in a study that suggested an alternative ferric reducing activity power (FRAP) assay [23].

The data presented in this study are useful for evaluating the reducing activity of each standard antioxidant compound separately but do not allow the distinction of each compound in complex samples. Moreover, it is not suitable to understand the individual contribution of any standard compound in order to find out the reducing capacity of a complex mixture. Even if the composition of a complex mixture is known the sum of response of several antioxidant standard solutions may not lead to the sum of these contributions in the mixture because chemical reactivity may be different due to synergistic or antagonistic effects. Nevertheless, the proposed method using the reduction reaction of Cu(II) to Cu(I) in BCA medium allows the spectrophotometric quantification of the reducing capacity of plant extracts, which are complex mixtures containing various antioxidants (especially polyphenols) available in different chemical forms.

### 2.2. Determination of the Reduction Capacity of Aqueous Extracts of Medicinal Plants with the Proposed Method

A calibration curve with AA standard solution was used to convert the A_558nm_ of the samples in AA_EC_. Assuming an AA theoretical concentration of 1.0 mg·mL^−1^ the A_558nm_ value is found using the equation A_558nm_ = *a* + *b* × C_AA_, in mg·mL^−1^. Replacing this A_558nm_ value in the equation of the calibration curve obtained with the plant extracts (A_558nm_ = *a* + *b* × C_DM_, in mg·mL^−1^) it was calculated the corresponding dry material (DM) concentration equivalent to a 1.0 mg·mL^−1^ AA solution.

Each analysis was performed in triplicate and the calculations were made taking into account the dilution of each extract. The mass of dry material responsible for the reducing capacity expressing in mg DM/g AA is shown in Table 2. Each analysis was performed in triplicate.

Considering the twelve plants analysed (Table 2) a significant correlation (r^2^ = 0.959) was found between the reduction capacity values obtained with the proposed method (mg DM/g AA) and with the DPPH reagent (g DM/g DPPH). DPPH method uses extracts obtained with organic solvents (acetone and methanol) and the proposed method (Cu(BCA)_2_^3−^ complexes) is an aqueous one. This difference could be responsible for the small discrepancy in the results obtained with these two methods.

It can be observed in Table 2 that the higher the reducing capacity the greater the total polyphenol content (TPC) of the extracts. In fact, the reducing capacity values obtained with the DPPH reagent (g DM/g DPPH) are proportional (r^2^ = 0.826; from DPPH EC_50_ *vs.* TPC) to TPC values obtained with the FC reagent (g PA/100 g DM).

Additionally, for this same set of samples (Table 2) the reduction capacity values obtained with the proposed method (mg DM/g AA) showed a better a correlation (r^2^ = 0.919; from Cu(BCA)_2_^3−^ *vs.* TPC) with the TPC values obtained with the FC reagent (g PA/100 g DM). This confirms that the reducing capacity of the extracts evaluated can be related to the polyphenolic content and the Cu(BCA)_2_^3−^ complexing activity can also be used to determine TPC as it was mentioned in our previous work [10].

**Figure 3:**
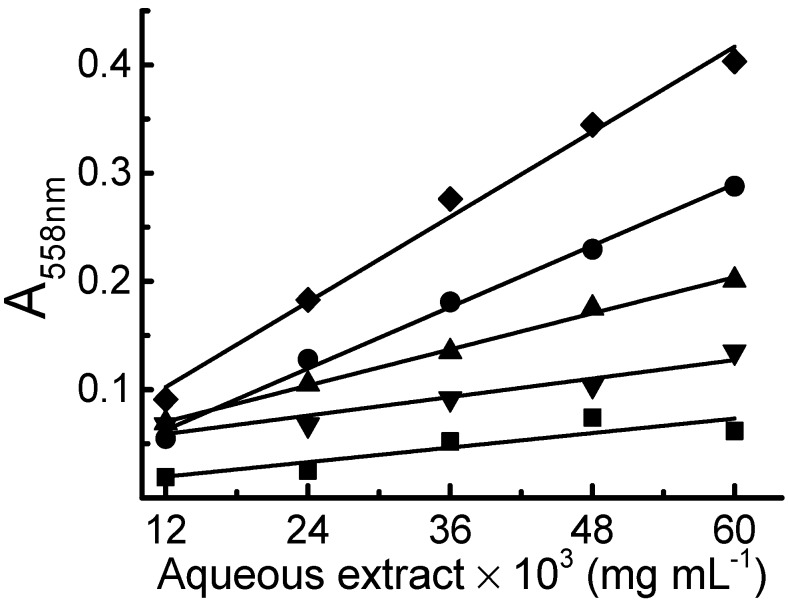
Addition of five aliquots of plant extracts in a solution containing 5.0 × 10^−4^ M Cu(II) + 1.5 × 10^−3^ M BCA + 0.8 M ammonium acetate. ■ = *Geissospermum leave* (Vell.) Miers; ▼ = *Annona muricata* L.; ▲ = *Bauhinia splendens* Kunth; ● = *Carapa guianensis* Aubl. And ♦ = *Salacia impressifolia* (Miers) A. C. Sm. A_558nm_ values taken using freshly prepared Cu(BCA)_2_^3−^ complex as reference solution.

Figure 3 shows the calibration curves obtained with the extracts of five plants analyzed (*Geissospermum laeve* (Vell.) Miers, *Carapa guianensis* Aubl., *Bauhinia splendens, Annona muricata* L. and *Salacia impressifolia*), which shows good linearity indicating that the reducing capacity of those plant extracts (in the specified concentration range) can be evaluated with the proposed reaction. Considering that all the extracts are at the same concentration it can be concluded that the greater the slope of the calibration curve the higher the reducing capacity of the extract.

### 2.3. Some Critical Considerations of the Proposed Method

The proposed method uses a spectrophotometer that is present in most laboratories, reagents which are not that expensive and the tests can be performed in approximately fifteen minutes. The ORAC method requires a fluorometer and needs fluorescent markers, which may not be available in analytical laboratories. Despite the fact that the ORAC method can be adapted to detect both hydrophilic and hydrophobic antioxidants, the long analysis time has been pointed out as a major drawback [1].

From the environmental point of view, the proposed method has the advantage of using the same aqueous extracts for measurements of the TPC (with FC reagent) and the reducing capacity (with Cu(BCA)_2_^3−^ complexes). In fact, the DPPH [23] and ABTS [24] methods, which are widely used for the determination of antioxidant capacity in plant samples, require organic solvents such as methanol and acetone.

**Table 2:** Reducing capacity and total polyphenol content of some Brazilian medicinal plants.

Plants	Common Brazilian Names	Use in Folk Medicine [25,26]	TPC (g PA/100 g DM)	Cu(BCA)_2_^3−^ (mg DM/g AA)	DPPH EC_50_ (g DM/g DPPH)
*Geissospermum laeve* (Vell.) Miers	Pau Pereira	Tonic properties; antifebrile	1.53 ± 0.06	22.2 ± 0.6	6.2 ± 2.3
*Schinus terebinthifolius* Raddi	Aroeira	Washing wounds; ulcers	5.57 ± 0.52	280 ± 15.7	94.8 ± 2.9
*Carapa guianensis* Aubl.	Andiroba	Bacterial infection; treatment of tumors	2.55 ± 0.19	96.2 ± 3.8	25.1 ± 1.0
*Bauhinia splendens* Kunth	Escada de Jabuti	Diabetes; treating obesity	1.42 ± 0.14	61.3 ± 3.7	19.7 ± 1.2
*Annona muricata* L.	Graviola	Anti-diarrheal; against spasms	1.90 ± 0.06	34.4 ± 0.6	8.4 ± 1.5
*Salacia impressifolia* (Miers) A. C. Sm.	Miraruíra	Muscle relaxant; rheumatism	3.52 ± 0.39	118 ± 4.0	34.3 ± 2.7
*Dipteryx odorata* (Aubl.) Willd.	Cumaru	Antispasmodic; moderator of the cardiac movements and breathing	1.40 ± 0.12	45.2 ± 3.9	12.2 ± 1.3
*Maytenus ilicifolia* Mart. Ex Reissek	Espinheira Santa	Ulcers; indigestion; chronic gastritis; dyspepsia	0.70 ± 0.04	15.8 ± 0.5	10.7 ± 5.0
*Stachytarpheta cayennensis* (Rich.) Vahl	Gervão	Anti-inflammatory; analgesic; gastroprotective	1.92 ± 0.05	41.3 ± 0.2	11.8 ± 1.8
*Hymenaea courbaril* L.	Jatobá	Cystitis; bronchitis; bladder infections; vermifuge	1.41 ± 0.19	32.9 ± 2.1	14.0 ± 0.4
*Cordiae calyculata* Vell.	Porangaba	Diuretic; treating obesity	1.30 ± 0.08	37.9 ± 1.3	8.8 ± 1.3
*Lippia grandis* Schauer	Salva de Marajó	Antimicrobial activity; treatment of liver diseases; stomach disease	2.40 ± 0.10	64.1 ± 2.9	7.5 ± 1.2

TPC is total polyphenol content quantified using the FC reagent (g pyrogallic acid/100 g dry material); Cu(BCA)_2_^3−^ is the reducing capacity expressed in ascorbic acid equivalents AA_EC_; DPPH EC_50_ is the antioxidant capacity using the DPPH reagent. Data represent average and standard deviation of at least three measurements.

Furthermore, the extraction step in DPPH method makes it more time-consuming compared to the suggested method. As the reduction of Cu(II) by antioxidants in BCA medium is fast, a flow injection procedure could be developed to reduce reagent consumption and consequently generate less waste.

However, if the analyte is a weak reducing agent (like some drugs) the reaction becomes slow and a Cu(II)/BCA light green precipitate is formed. In this case is necessary to perform the procedure under heating and in a micellar medium to accelerate this reaction [11,12].

Considering the hazardous aspects of the reaction used it is necessary to mention that the toxicity of BCA was not fully studied yet, so it should be properly disposed of. In a previous work a simple way to recycle and reuse this ligand for qualitative purposes was described [11].

## 3. Experimental Section 

### 3.1. Apparatus

All absorbance measurements were recorded on a HP UV 8453 spectrophotometer (Agilent Technologies, Santa Clara, CA, USA) using a 1.0 cm optical path length glass cell.

### 3.2. Reagents and Solutions

Reverse osmosis water (Quimis Q842-210, Diadema, Brazil) was used in all solutions except when another solvent is indicated. All reagents used were of analytical grade. Disodium salt of 4,4′-dicarboxy-2,2′-biquinoline acid (Na_2_BCA, > 99%, FW 388.3 g·mol^−1^), ammonium acetate (NH_4_(H_3_C-COO), 98%, FW 77.08 g·mol^−1^) and ascorbic acid (AA, C_6_H_8_O_6_, 99%, 176.12 g·mol^−1^) were from Sigma-Aldrich (São Paulo, Brazil). Copper(II) perchlorate, Cu(ClO_4_)_2_, 2.328 M solution was synthesized and standardized as described previously [12]. Information on the preparation and dilution of all reagents can be found in the Supplementary Material.

### 3.3. Preparation of Aqueous Extracts of Medicinal Plants

The method described in the Brazilian Pharmacopoeia [15] for preparing aqueous extracts was used for quantifying the polyphenol content and to determine the total reducing capacity with the Cu(I)/BCA complexes [10,14,23].

### 3.4. Preparation of Extracts for Determining the Reduction Capacity with DPPH

This procedure was also described elsewhere [13,14,23] and is in the Supplementary Material.

### 3.5. Total Polyphenol Content Quantification with the FC Reagent

For quantifying the total polyphenol content the method described in the Brazilian Pharmacopoeia [15] was slightly modified, using a 10-fold reduction in the amount of all reagents and is also described in the Supplementary Material.

### 3.6. Determination of the Total Reducing Capacity of Plant Extracts with DPPH Reagent

The DPPH method for determining the total reducing capacity was carried out as described elsewhere [14,23]. The values found correspond to the dry material of plant extracts required to reduce the initial DPPH concentration by 50% (EC_50_). The EC_50_ results are expressed as g dry material (DM)/g DPPH.

### 3.7. Proposed Method for Reducing Capacity Quantification

#### 3.7.1. Calibration Curve with Ascorbic Acid Standard Solution

A typical calibration curve was obtained by mixing 250 μL of 1.0 × 10^−2^ M Cu(II) solution and 2.0 mL of 2.0 M ammonium acetate buffer solution (pH 7.0) in eight 5.0 mL volumetric flasks. After homogenization aliquots (200–900 μL) of 1.0 × 10^−3^ M (0.0177 mg·mL^−1^) AA standard solution and 250 μL of 3.0 × 10^−2^ M BCA solution were transferred to the flasks and volume completed with water. The AA final concentration varied from (7.05 to 31.7) × 10^−3^ mg·mL^−1^. A_558nm_ values were taken using as reference a freshly prepared solution above described (without AA). With this calibration curve (A_558nm_ *vs.* C_AA_, in mg·mL^−1^) it was obtained a straight-line equation (A_558nm_ = *a* + *b* × C_AA_), where *a* and *b* are the y-intercept and the slope, respectively. This calibration curve was performed just before each sample analysis.

#### 3.7.2. Calibration Curves with Standard Antioxidant Compounds

These calibration curves were obtained by transferring aliquots (100–800 µL) of each antioxidant compound solution (1.0 × 10^−4^ to 5.3 × 10^−2^ M) to several 5.0 mL volumetric flasks containing 250 μL of 1.0 × 10^−2^ M Cu(II) solution and 2.0 mL of 2.0 M ammonium acetate buffer solution (pH 7.0). After homogenization 250 μL of 3.0 × 10^−2^ M BCA solution was diluted with water. Then, the same procedure described above for the AA calibration curve was performed. All A_558nm_ values were taken using as reference a freshly prepared solution as described above but without antioxidant compounds.

#### 3.7.3. Calibration Curve with Aqueous Plant Extracts of Medicinal Plants

Aliquots of the aqueous extract (100 to 500 μL) were transferred to five 5.0 mL volumetric flasks containing 250 μL of 1.0 × 10^−2^ M Cu(II) solution and 2.0 mL of 2.0 M ammonium acetate buffer solution (pH 7.0). After homogenization 250 μL of 3.0 × 10^−2^ M BCA solution were added and volume completed with water. The dry material concentration, C_DM_, in each flask was expressed in mg·mL^−1^. All A_558nm_ values were taken using freshly prepared solution (without the aqueous extracts) as reference. A calibration curve (A_558nm_ *vs.* C_DM_ in mg·mL^−1^) was obtained yielding a straight-line equation (A_558nm_ = *a* + *b* × C_DM_), where *a* and *b* are the *y*-intercept and the slope, respectively.

## 4. Conclusions

The proposed procedure to measure the total reducing capacity was successfully applied to plant extracts, indicating that it can be used in other aqueous samples of vegetable origin such as tea, beer, wine and fruit juices. As the spectrophotometer is simple to operate, and available in most laboratories, and the ligand used (4,4′-dicarboxy-2,2′-biquinoline acid) is not very expensive, the suggested method can be adapted to routine analysis. It is easy to perform and because the reaction is fast, it can be also used in flow injection systems. This method can be employed to determine the total reducing capacity of biological samples as serum, follicular fluid, tears and urine. As the reaction is not conducted in organic solvents (e.g., acetone or methanol) the proposed method is more attractive from an environmental point of view.

## Supplementary Materials

Supplementary materials can be accessed at: http://www.mdpi.com/1420-3049/20/12/19855/s1.
