# Supplementary material for: A Comprehensive Study of the Use of Cu(I)/4,4’-Dicarboxy-2,2’-biquinoline Complexes to Measure the Total Reducing Capacity: Application in Herbal Extracts
Source: Molecules. 2015 Dec 14;20(12):22411–21. doi: 10.3390/molecules201219855 (PMC6331943; doi:10.3390/molecules201219855)
Supplement: Supplementary file 1 [file molecules-20-19855-s001.pdf]

Supplementary Materials: A Comprehensive Study of the Use of the Cu(I)/4,4′-Dicarboxy-2,2′-biquinoline Complexes to Measure the Total Reducing Capacity: Application in Herbal Extracts

Hariane R. Manoel and Horacio D. Moya

Reagents and solutions

Disodium salt of 4,4′-dicarboxy-2,2′-biquinoline (Na_2BCA, > 98%, FW 388.3 g·mol^{-1}, Sigma-Aldrich, São Paulo, Brazil) 3.0 × 10^{-2} M solution, was prepared by dissolving 0.5825 g in a 50 mL volumetric flask.

Copper(II) perchlorate, $Cu(ClO_4)_2$, 2.328 M solution was synthesized and standardized as described in previous studies [10]. A 1.0 × 10^{-2} M diluted solution used was prepared accurately.

Ammonium acetate ($NH_4(H_3C\text{-}COO)$, 98%, FW 77.08 g·mol^{-1}, Sigma-Aldrich) 2.0 M solution, was prepared by dissolving 77.08 g in a 500 mL volumetric flask and used as pH 7.0 buffer solution.

Ascorbic acid (AA, $C_6H_8O_6$, 99%, FW 176.12 g·mol^{-1}, Sigma-Aldrich) 1.0 × 10^{-3} M (0.177 mg·mL^{-1}) standard solution was prepared daily by dissolving 0.1761 g in a 100 mL volumetric flask.

Tannic acid ($C_{76}H_{52}O_{46}$, 99%, FW 1701.2 g·mol^{-1}), gallic acid ($C_7H_6O_5$, 98%, FW 170.12 g·mol^{-1}), phloroglucinol ($C_6H_6O_3$, 99%, FW 126.1 g·mol^{-1}), pyrogallic acid ($C_6H_6O_3$, 99%, FW 126.1 g·mol^{-1}), 1,2,4-benzenotriol ($C_6H_6O_3$, 99%, FW 126.1 g·mol^{-1}), pyrocatechol ($C_6H_6O_2$, ≥ 99%, FW 110.1 g·mol^{-1}), hydroquinone ($C_6H_6O_2$, ≥ 99%, FW 110.1 g·mol^{-1}), resorcinol ($C_6H_6O_2$, ≥ 99%, FW 110.1 g·mol^{-1}) and phenol (C_6H_6O, ≥ 99%, FW 94.11 g·mol^{-1}) 1.0 × 10^{-4} to 5.3 × 10^{-2} M stock solutions (all from Labsynth, São Paulo, Brazil) were prepared by dissolution in water.

(−)-Epigallocatechingallate ($C_{22}H_{18}O_{11}$, ≥ 98%, FW 458.4 g·mol^{-1}), sinapic acid ($C_{11}H_{12}O_5$, ≥ 98%, FW 224.21 g·mol^{-1}), 2,3,4-THB (2,3,4-trihydroxybenzoic acid, $C_7H_6O_4$, 97%, FW 170.12 g·mol^{-1}), vanillin ($C_8H_8O_3$, 99%, FW 152.2 g·mol^{-1}), vanillic acid ($C_8H_8O_4$, > 97%, FW 168.15 g·mol^{-1}) and 4-hydroxyphenylacetic acid ($HOC_6H_4CH_2CO_2H$, 98%, FW 152.15 g·mol^{-1}) 1.0 × 10^{-4} to 1.0 × 10^{-3} M stock solutions (all from Sigma-Aldrich) were prepared by dissolution in water.

Caffeic acid ($C_9H_8O_4$, > 98%, FW 180.2 g·mol^{-1}, Sigma-Aldrich) and ferulic acid ($C_{10}H_{10}O_4$, 99%, FW 194.2 g·mol^{-1}, Sigma-Aldrich) 1.0 × 10^{-3} M stock solutions were prepared in water with addition of six drops of 0.20 M NaOH solution.

p-coumaric acid ($C_9H_8O_3$, ≥ 98%, FW 164.2 g·mol^{-1}, Sigma-Aldrich) 1.0 × 10^{-3} M stock solution was prepared in water with addition of two drops of 0.20 M NaOH solution.

Quercetin ($C_{15}H_{10}O_7$, 98 % HPLC, FW 302.2 g·mol^{-1}, Sigma-Aldrich, São Paulo, Brazil) 1.0 × 10^{-3} M stock solution was prepared by dissolving 0.03283 g in 50:50 *v/v* ethanol:water. A 1.0 × 10^{-4} M solution was obtained by diluting in water with addition of one drop of 0.20 M NaOH solution.

Rutin ($C_{27}H_{30}O_{16}$, 95% HPLC, FW 610.5 g·mol^{-1}, Sigma-Aldrich) 1.0 × 10^{-3} M stock solution, was prepared by dissolving 0,0665 g in 30 mL ethanol (CH_3CH_2OH, 99.6%, FW 46.07 g·mol^{-1}, d = 0.789 g·cm^{-3}, Synth, São Paulo, Brazil), adding five drops 0.20 M NaOH solution and completing the volume with water. A 1.0 × 10^{-4} M solution was prepared by accurate dilution in water.

Trolox ($C_{14}H_{18}O_4$, 97 %, FW 250.29 g·mol^{-1}, Sigma-Aldrich) 4.0 × 10^{-4} M stock solution was prepared by mixing 8.0 mL ethanol (CH_3CH_2OH, 99.6%, FW 46.07 g·mol^{-1}, d = 0.789 g·cm^{-3}, Synth) and 0.20 mL NaOH 0.20 M in a 100.0 mL volumetric flask. The solution remained in the ultrasound bath for 10 min and then the volume was completed with water.

β-carotene ($C_{40}H_{56}$, ≤ 95%, FW 536.87 g·mol^{-1}, Sigma-Aldrich) 1.0 × 10^{-3} M stock solution was prepared by dissolving 0.0272 g in 40:10 (*v/v*) acetone:water in a 50 mL volumetric flask.

The Folin-Ciocalteu reagent was prepared as described in Brazilian Pharmacopoeia [15] as follow: 20 g of sodium tungstate ($Na_2WO_4·2H_2O$, ≥ 99%, FW 329.85 g·mol^{-1}, Vetec, Rio de Janeiro Brazil, 4.0 g of phosphomolybdic acid ($H_3Mo_{12}O_{40}P·H_2O$, ≥ 99.9%, FW 1825.25 g·mol^{-1}, Sigma-Aldrich), and 10 mL of phosphoric acid (H_3PO_4, 85%, FW 98.00 g·mol^{-1}, Merck, São Paulo, Brazil) were dissolved

in 150 mL of water. This solution was heated under reflux for 2 h and after cooling at room temperature it was diluted with water to 200 mL.

Sodium carbonate (Na_2CO_3, ≥ 99 %, FW 105.99 g·mol^{-1}, Vetec, Rio de Janeiro, Brazil) 10% (*m*/*v*) solution was prepared in water.

A 50% (*v*/*v*) methyl alcohol (CH_3COH, 99.8%, FW 32.04 g·mol^{-1}, d = 0.792 g·cm^{-3}, Merck) and a 70 % (*v*/*v*) acetone (CH_3OCH_3, ≥ 99.5%, FW 58.08 g·mol^{-1}, d = 0.79 g·cm^{-3}, Merck) solutions were prepared in water.

A 0.0237 mg·mL^{-1} DPPH (2,2-diphenyl-1-picrylhydrazyl, $C_{18}H_{12}N_5O_6$, FW 394.32 g·mol^{-1}, Sigma-Aldrich) solution was prepared daily by dissolving 2.4 mg in methyl alcohol (CH_3COH, 99.8%, FW 32.04 g·mol^{-1}, d = 0.792 g·cm^{-3}, Merck) in a 100.0 mL volumetric flask.

Preparation of Aqueous Extracts of Medicinal Plants According the Brazilian Pharmacopoeia

0.75 g of dry material (leaf, branch or root) of any plant was transferred to a 250 mL erlenmeyer containing 150 mL of water and after homogenization it was maintained on a water bath (80–90 °C; 30 min). After cooling, the mixture was transferred to a 250 mL volumetric flask which was completed with water. After the plant material decanted the solution was filtered through filter paper discarding the first 50 mL. Next 5.0 mL of the filtrate above were transferred to a 25 mL volumetric flask which was completed with water and then used in measurements [23].

Preparation of Samples Extracts for Determining the Reduction Capacity with DPPH

Dry plant material (1.00 g) was transferred to a 100 mL beaker which was added 40 mL of a 50% (*v*/*v*) methyl alcohol aqueous solution. After mixing the mixture was allowed to stand (25 °C; 1 h). This mixture was then centrifuged (15 min; 15000 rpm) and the supernatant was transferred to a 100 mL volumetric flask. Next, 40 mL of a 70% (*v*/*v*) acetone aqueous solution were added in the centrifuged. After homogenization the mixture was also allowed to stand (25 °C; 1 h). Then, this new mixture was centrifuged (15 min; 15000 rpm) and the supernatant obtained was transferred to the same 100 mL volumetric flask. Water was added to complete the volume [11,15,16].

Total Polyphenol Content Quantification with the FC Reagent

The procedure for quantifying the total polyphenol content described in the Brazilian Pharmacopoeia was slightly modified using the 10-fold reduction in the amount of all reagents [23]. So, 5.0 mL of the filtrate (see *Preparation of aqueous extracts of medicinal plants*) were transferred to a 25 mL volumetric flask and completed with water.

Firstly, a calibration curve was raised by mixing aliquots of 100 to 800 µL of a 1.0 × 10^{-4} M pyrogallic acid (PA) standard solution with 200 µL of FC reagent and completed with a 10% Na_2CO_3 solution in a 5.0 mL volumetric flask.

The multiple standard addition method was used for quantification of all samples transferring 100–500 µL of aqueous extracts (depending of the species) to five 5.0 mL volumetric flasks and then added 200 µL of FC reagent. In four out of five volumetric flasks were added aliquots 150, 200, 250 and 300 µL of 1.0 × 10^{-4} M PA solution and the flasks completed with 10% Na_2CO_3 solution.

In both curves (calibration and multiple standard additions) the absorbance was measured at 715 nm (A_{715nm}) after 30 min using water as reference solution.
